# The prevalence of fatigue among Chinese nursing students in post-COVID-19 era

**DOI:** 10.7717/peerj.11154

**Published:** 2021-04-13

**Authors:** Shou Liu, Hai-Tao Xi, Qian-Qian Zhu, Mengmeng Ji, Hongyan Zhang, Bing-Xiang Yang, Wei Bai, Hong Cai, Yan-Jie Zhao, Li Chen, Zong-Mei Ge, Zhiwen Wang, Lin Han, Pan Chen, Shuo Liu, Teris Cheung, Brian J. Hall, Feng-Rong An, Yu-Tao Xiang

**Affiliations:** 1 Department of Public Health, Medical College, Qinghai University, Xining, Qinghai province, China; 2 Centre for Cognitive and Brain Sciences, University of Macau, Macao, China; 3 Institute of Advanced Studies in Humanities and Social Sciences, University of Macau, Macao, China; 4 Nursing College, Jilin University, Changchun, Jilin province, China; 5 School of Nursing, Capital Medical University, Beijing, China; 6 The National Clinical Research Center for Mental Disorders & Beijing Key Laboratory of Mental Disorders Beijing Anding Hospital & the Advanced Innovation Center for Human Brain Protection, Capital Medical University, School of Mental Health, Beijing, China; 7 School of Nursing, Peking University, Beijing, China; 8 School of nursing, Lanzhou University, Lanzhou, Gansu province, China; 9 School of Health Sciences, Wuhan University, Wuhan, Hubei province, China; 10 School of Nursing, Hong Kong Polytechnic University, Hong Kong, China; 11 School of Global Public Health, New York University, New York, NY, USA

**Keywords:** Fatigue, Quality of life, Nursing students, COVID-19 pandemic

## Abstract

**Background:**

Due to the COVID-19 outbreak, all teaching activities in nursing schools were suspended in China, and many nursing students were summoned to work in hospitals to compensate for the shortage of manpower. This study examined the prevalence of fatigue and its association with quality of life (QOL) among nursing students during the post-COVID-19 era in China.

**Methods:**

This was a multicenter, cross-sectional study. Nursing students in five Chinese universities were invited to participate. Fatigue, depressive and anxiety symptoms, pain and QOL were measured using standardized instruments.

**Results:**

A total of 1,070 nursing students participated. The prevalence of fatigue was 67.3% (95% CI [64.4–70.0]). Multiple logistic regression analysis revealed that male gender (*P* = 0.003, OR = 1.73, 95% CI [1.20–2.49]), and being a senior nursing student (second year: OR = 2.20, 95% CI [1.46–3.33], *P* < 0.001; third year: OR = 3.53, 95% CI [2.31–5.41], *P* < 0.001; and fourth year OR = 3.59, 95% CI [2.39–5.40], *P* < 0.001) were significantly associated with more severe fatigue. In addition, moderate economic loss during the COVID-19 pandemic (OR = 1.48, 95% CI [1.08–3.33], *P* < 0.015; compared to low loss), participants with more severe depressive (OR = 1.48, 95% CI [1.22–1.78], *P* < 0.001) and anxiety symptoms (OR = 1.12, 95% CI [1.05–1.20], *P* = 0.001), and more severe pain (OR = 1.67, 95%CI [1.46–1.91], *P* < 0.001) were significantly associated with reported more severe fatigue. After controlling for covariates, nursing students with fatigue had a lower overall QOL score compared to those without (*F*_(1, 1070)_ = 31.4, *P* < 0.001).

**Conclusion:**

Fatigue was common among nursing students in the post-COVID-19 era. Considering the negative impact of fatigue on QOL and daily functioning, routine physical and mental health screening should be conducted for nursing students. Effective stress-reduction measures should be enforced to assist this subpopulation to combat fatigue and restore optimal health.

## Introduction

Fatigue refers to abnormal exhaustion following normal activities (Cavanaugh, 2002; Shapiro et al., 2005). Fatigue is associated with lifestyle factors (e.g., physical exertion, lack of sleep, use of antidepressants), physical health problems (e.g., anemia, autoimmune disorders, and chronic obstructive pulmonary disease), and mental health problems (e.g., sleep disorders, anxiety, and depression) (De Venter et al., 2017; Friedberg et al., 2016). All of these factors could lead to additional detrimental outcomes such as headache, faintness, shortness of breath, and increased risk of suicidality (Zhu, Han & Li, 2019).

The prevalence of fatigue varies in different populations. For instance, the prevalence of fatigue ranged from 15% to 30% in teenagers (Findlay, 2008; Ghandour et al., 2004); 11.9% in adults, with 8.5% in men, and 14.9% in women (Wendt et al., 2019). The prevalence of fatigue was usually more common in certain subpopulations. For instance, the Australian Medical Association found that out of 716 doctors, 53% were at higher risk of fatigue whilst on duty (Australian Medical Association, 2017). In another study, around 85% of patients with head and neck cancer experienced fatigue (Bossi et al., 2019). In addition, college students, particularly those enrolled in health-related subjects, often suffered from fatigue (Dol, 2016; Pallant, Sullivan & Kaluzny, 2020; Shim et al., 2019). For example, one study found that the prevalence of fatigue was 16.5% among medical students (Tanaka et al., 2008), while the corresponding figure was even higher among nursing students (39.1%) (De Moraes Amaducci, De Correa Mota & De Mattos Pimenta, 2010).

Coronavirus Disease 2019 (COVID-19) was first reported in Wuhan, China in December, 2019 (Huang et al., 2020) and then found in more than 200 countries and territories (World Health Organization, 2020). Since April 2020, COVID-19 has been well-contained in China (National Health Commission of China, 2020). To lower the risk of contagion between students, the spring semester was postponed in all universities in China in early 2020. Further, all classroom teaching was suspended, and replaced by online teaching and learning (Xinhuanet, 2020a, 2020b). Due to lockdown measures, outdoor/physical activities were prohibited in many areas of China. In addition to sudden changes of traditional face-to-face learning modes, students were exposed to high level of academic stress (Brooks et al., 2020; Wang et al., 2020a), which may trigger negative health outcomes including fatigue (Elhai et al., 2020; Király et al., 2020; Miao et al., 2020a, 2020b). Compared to those enrolling in other non-health related subjects, students in health-related majors, such as nursing, may be at higher risks of fatigue due to higher curriculum demand and academic workload from the faculty of nursing.

In order to reduce the likelihood of negative health outcomes caused by fatigue, it is important to understand its prevalence and associated factors. To date, however, fatigue among nursing students in the post- COVID-19 era has not been investigated. Therefore, the aims of this study were to: (1) examine the prevalence of fatigue among nursing students in the post COVID-19 era in China and (2) explore the association between fatigue and quality of life (QOL) among nursing students.

## Methods

### Participants and study settings

This was a multicenter, cross-sectional study conducted between September 14 and October 7, 2020 across five university nursing schools (Peking University, Capital Medical University, Jilin University, Lanzhou University, and Wuhan University) in China. In order to avoid contagion during the COVID-19 pandemic, face-to-face interviews were not plausible. Consistent with other studies (Bo et al., 2020; Ma et al., 2020), data were collected using the Questionnaire Star Application embedded in WeChat, which is a widely used social communication application with over 1 billion users in China. WeChat had been used as a teaching tool in the participating nursing schools, therefore, all students were WeChat users. Nursing students in the participating nursing schools were consecutively invited to participate in this study, and those who electronically signed the online written informed consent could access the assessment. To be eligible, participants were (1) undergraduate nursing students, (2) aged between 15 and 28 years, (3) able to understand the content of the assessments, and (4) able to provide written informed consent. The study protocol (No:2020-10) was approved by the Institutional Review Board (IRB) at Beijing Anding Hospital of Capital Medical University and all collaborating university nursing schools.

### Measurement tools

Basic socio-demographic data, such as gender, age, year of study, perceived economic status were collected based on self-report. COVID-19 related experiences were asked using standardized questions, including (1) whether they were volunteers in clinical settings during the COVID-19 pandemic; (2) whether they had negative experiences (e.g., such as verbal abuse and threats) during the COVID-19 pandemic; (3) whether they experienced economic loss during the COVID-19 pandemic; (4) whether they used social media frequently to obtain relevant information during the COVID-19 pandemic; and (5) economic status and perceived health status were also asked using standardized questions.

Fatigue was measured using the numeric rating scale (NRS), scoring from “0” (not suffering from fatigue) to “10” (unbearable suffering from fatigue) (Berger et al., 2010). Higher scores indicated more severe fatigue, and a score of ≥4 was considered “clinically relevant fatigue” (“having fatigue” hereafter) (Oldenmenger et al., 2013). Another NRS was adopted to evaluate severity of overall body pain (pain hereafter) (Haefeli & Elfering, 2006), which was scored from “0” (no pain) to “10” (worst pain imaginable), with a higher score indicating more severe pain (Li, Herr & Chen, 2009; Li, Liu & Herr, 2007; Liu & Li, 2004).

The Chinese version of the Patient Health Questionnaire (PHQ-2) was used to measure depressive symptoms (Chen, Sheng & Qu, 2015; Kroenke, Spitzer & Williams, 2001). Each item scored from 0 (not at all) to 3 (nearly every day). The total score ranged from 0 to 6, with a higher score representing more severe depressive symptoms. The Chinese version of the Generalized Anxiety Disorder scale seven items (GAD-7) was used to assess anxiety symptoms. Each item scored from 0 (not at all) to 3 (almost every day), with a higher score indicating more severe anxiety symptoms (He et al., 2010; Spitzer et al., 2006). QOL was measured using the first two items on overall QOL of the World Health Organization Quality of Life-brief version (WHOQOL-BREF) (Fang & Hao, 1999; Harper, Power & The WHOQOL group, 1998; Xia et al., 2012), with higher scores indicating greater QOL.

### Statistical analysis

Data were analyzed using the IBM Statistical Package for Social Science (SPSS) program, version 24.0. The comparisons between nursing students with and without fatigue were conducted using two independent samples *t* tests, Mann-Whitney U Tests, and Chi-square tests, as appropriate. Analysis of covariance (ANCOVA) was conducted to examine the independent association between fatigue and QOL, after adjusting for variables with significant group differences in univariate analyses. Binary logistic regression analysis with the “enter” method was performed to test the independent correlates of fatigue, with fatigue as the dependent variable, and those with significant group differences in the univariate analyses as independent variables. Significance level was set at 0.05 (two-tailed).

## Results

Altogether, 1,121 nursing students were consecutively invited to participate in this study; of whom, 1,070 met the study criteria and completed the assessment, yielding a response rate of 95.5%. The prevalence of fatigue was 67.3% (95% CI [64.4–70.0]). There were significant differences between fatigue and no fatigue groups in terms of gender, age, year of study, economic loss during COVID-19 pandemic, financial perception, health perception, and the PHQ-2, GAD-7, and pain total scores (Table 1). After controlling for covariates, nursing students with fatigue had lower QOL (*F*_(1, 1070)_ = 31.4, *P* < 0.001) than those without.

**Table 1:** Socio-demographical and scale’ scores of nursing students.

Variables	Total		Non Fatigue		Fatigue		Univariate analyses		
	(*N* = 1,070)		(*N* = 350)		(*N* = 720)				
	*N*	%	*N*	%	*N*	%	*χ*^2^	df	*P*
Male gender	265	24.8	63	18.0	202	28.1	12.78	1	**<0.001**
Rural residence	457	42.7	145	41.4	312	43.2	0.35	1	0.555
Only Child	457	42.7	147	42.0	310	43.1	0.11	1	0.742
Year of Study							64.11	3	<0.001
First year	287	26.8	147	42.0	140	19.4			
Second year	237	22.1	72	20.6	165	22.9			
Third year	249	23.3	60	17.1	189	26.3			
Fourth year	297	27.9	71	20.3	226	31.4			
Being volunteer during COVID-19 pandemic	231	21.6	66	18.9	165	22.9	2.29	1	0.130
Having negative experiences during COVID-19 pandemic	188	17.6	51	14.6	137	19.0	3.23	1	0.073
Economic loss during COVID-19 pandemic							18.35	**2**	**<0.001**
Not or mild	444	41.5	177	50.6	267	37.1			
Moderate	557	52.1	157	44.9	400	55.6			
Great	69	6.4	16	4.6	53	7.4			
Frequent use of social media during COVID-19 pandemic	778	72.7	252	72.0	526	73.1	0.13	1	0.716
Perceived economic status							8.90	**2**	**0.012**
Poor	218	20.4	63	18.0	155	21.5			
Fair	776	72.5	251	71.7	525	72.9			
Rich	76	7.1	36	10.3	40	5.6			
Perceived health status							45.70	**2**	**<0.001**
Poor	23	2.1	4	4.1	19	2.6			
Fair	449	42.0	99	28.3	350	48.6			
Good	598	55.9	247	70.6	351	48.8			
	Mean	SD	Mean	SD	Mean	SD	*t*/*Z*	df	*P*
Age (years)	19.7	1.4	19.4	1.5	19.9	1.4	5.55	1068	**<0.001**
Fatigue total	4.8	2.1	2.5	0.7	6.0	1.5	51.48	1068	**<0.001**
PHQ-2 total	1.0	1.2	0.5	0.8	1.3	1.3	10.76	--^a^	**<0.001**
GAD-7 total	3.1	3.9	1.4	2.5	4.0	4.2	11.09	--^a^	**<0.001**
Pain total	2.4	1.8	1.6	1.0	2.8	1.9	9.76	--^a^	**<0.001**
QOL total	6.7	1.5	7.5	1.3	6.4	1.5	12.58	1068	**<0.001**

**Notes:**

a Mann-Whitney U test.

Bolded values: <0.05.

COVID-19, Coronavirus Disease 2019; df, degree of freedom PHQ-2, the 2-item Patient Health Questionnaire; QOL, quality of life; GAD-7, 7-item Generalized Anxiety Disorder; SD, standard deviation.

Multiple logistic regression analysis revealed that men (Odds Ratio (OR) = 1.73, 95% CI [1.20–2.49], *P* = 0.003), students in their 2nd (OR = 2.20, 95% CI [1.46–3.33], *P* < 0.001), 3rd (OR = 3.53, 95% CI [2.31–5.41], *P* < 0.001) and 4th year (OR = 3.59, 95% CI [2.39–5.40], *P* < 0.001; compared to students in their first year), moderate economic loss during the COVID-19 pandemic (OR = 1.48, 95% CI [1.08–3.33], *P* = 0.015; compared to low loss), more severe depressive (OR = 1.48, 95% CI [1.22–1.78], *P* < 0.001), and anxiety symptoms (OR = 1.12, 95% CI [1.05–1.20], *P* = 0.001), and more severe pain (OR = 1.67, 95% CI [1.46–1.91], *P* < 0.001) were significantly associated with more severe fatigue (Table 2).

**Table 2:** Independent correlates of fatigue by multiple logistic regression analysis.

Variables	Multiple logistic regression analysis		
	*P*	OR	95% CI
Male gender	**0.003**	1.73	[1.20–2.49]
Year of study			
First year	–	1.0	–
Second year	**<0.001**	2.20	[1.46–3.33]
Third year	**<0.001**	3.53	[2.31–5.41]
Fourth year	**<0.001**	3.59	[2.39–5.40]
Economic loss during COVID-19 pandemic			
Not or mild	–	1.0	–
Moderate	**0.015**	1.48	[1.08–2.02]
Great	0.352	1.41	[0.68–2.91]
Perceived economic status			
Poor	–	1.0	–
Fair	0.100	1.41	[0.94–2.13]
Rich	0.495	1.26	[0.65–2.44]
Perceived health status			
Poor	–	1.0	–
Fair	0.151	2.70	[0.70–10.50]
Good	0.312	2.02	[0.52–7.86]
PHQ-2 total	**<0.001**	1.48	[1.22–1.78]
GAD-7 total	**0.001**	1.12	[1.05–1.20]
Pain total	**<0.001**	1.67	[1.46–1.91]

**Notes:**

Bolded values: <0.05.

CI, confidential interval; OR, odds ratio; PHQ-2, the 2-item Patient Health Questionnaire; QOL, quality of life; GAD-7, 7-item Generalized Anxiety Disorder Scale. There was collinearity between age and grade, therefore age was not entered in the model as an independent variable.

## Discussion

This study examined the prevalence of fatigue among nursing students in post-COVID-19 era. We found that 67.3% of nursing students reported fatigue, which is almost double the prevalence of fatigue (36%) in qualified nurses on shift work assessed by the Occupational Fatigue Exhaustion Recovery scale (Geiger-Brown et al., 2012). Our finding was similar to the corresponding figure (73.7%) in frontline staff (including doctors, nurses, police officers, volunteers, community workers, and journalists) during COVID-19 outbreak in China as measured by the Fatigue Self-Assessment Scale (Teng et al., 2020). In contrast, the level of fatigue among medical students was relatively low (13.8%) before the COVID-19 outbreak (Abdali, Nobahar & Ghorbani, 2020). Owing to different measurement tools on fatigue, direct comparisons between studies should be interpreted with caution.

Fatigue appeared to be common among nursing students in the post-COVID-19 era and this can be attributed to several reasons. First, previous studies found that fatigue among students who majored in health-related subjects was usually related to poor academic performance and related problems, such as absenteeism, and having a sedentary lifestyle (e.g., lack of physical exercise) (Cruz et al., 2018). Sudden shifting from traditional classroom learning to online learning coupled with limited outdoor physical activities during the COVID-19 outbreak in China may have led to poorer academic performance, and increased absenteeism, which is often linked with sedentary lifestyle, and this in turn may have led to more fatigue among nursing students. Second, many nursing and medical students served as volunteers in clinical settings during the COVID-19 outbreak. Persistent high levels of stress and anxiety (Cao et al., 2020) at work could further exacerbate the risk of fatigue (Abdali, Nobahar & Ghorbani, 2020; Doerr et al., 2015; Nijrolder, Van der Horst & Van der Windt, 2008). In addition, potential risk of susceptibility to COVID-19 infection on top of a heavy clinical workload may have also escalated the risk of fatigue amidst the COVID-19 outbreak. Third, daily infection precautionary measures at work (e.g., face mask wearing, frequent hand-washing, full gear personal protection equipment adherence), reduced social etiquette practices (e.g., shaking hands) and social distancing, could lead to boredom (Miao et al., 2020b), anxiety, frustration (Aristovnik et al., 2020), and mental fatigue.

In this study, we found that male students were more likely to report fatigue than their female counterparts. In China, nursing students are predominantly women. In traditional Chinese culture, men have been ascribed the social status of “pillars” within the family and in the society; therefore, they were often expected to be responsible for more heavy tasks and challenges than women in public health crisis situations (e.g., COVID-19 outbreak). In addition, female students who major in health-related subjects usually have a better academic performance than male students (Alzahrani, Soo Park & Tekian, 2018; Voyer & Voyer, 2014). Such gender differences in academic performance suggests that female students may adapt better than male students in the switching of learning modes. These social and educational factors could result in greater fatigue in male students. Similar to previous findings (Labrague & Ballad, 2020), we found that the 2nd (OR = 2.20), 3rd (OR = 3.53) and 4th year students (OR = 3.59) were more likely to report fatigue than 1st year students. Senior nursing students receive more crisis response and medical training compared to junior students. As such, they usually undertook a greater responsibility in combating the COVID-19 outbreak, which possibly explained the differences in the level of fatigue between years of study.

As expected, more severe fatigue was associated with greater economic loss, more severe depressive and anxiety symptoms, and more severe pain among nursing students in this study. Greater economic loss could lead to psychological distress, which may in turn increase the risk of fatigue. Similar findings were found in university students (Shim et al., 2019) before and during COVID-19 outbreak (Verma, 2020; Wang et al., 2020b). The relationship between fatigue and depression/anxiety were bidirectional (Thorsteinsson et al., 2019) (i.e., fatigue could increase the risk of depression and anxiety, and vice versa). Consistent with previous findings (Kaasa et al., 1999; Yoon et al., 2019), in this study more severe pain was associated with a higher risk of fatigue in nursing students. Pain is defined as an unpleasant sensory and emotional experience usually associated with actual or potential tissue damage (Raja et al., 2020) caused by internal and/or external factors (e.g., cold, heat, physical pressure and lesions). Adjustment mechanisms in the human body attempt to relieve pain through the central brain feedback system (Mauger, 2013; Rainville, 2002). If the predisposing factors cannot be addressed and remain chronic, the adjustment/restoration system will be out of balance and the body will be fatigued (Aaronson et al., 1999; Sharpe & Wilks, 2002).

Similar to previous findings (Kratz et al., 2017; Nunes et al., 2017), we found that nursing students with fatigue had a lower overall QOL than those without. As a widely used health outcome measure, QOL is closely associated with the interactions between protective factors (e.g., better social support) and risk factors (e.g., physical distress) (Hatoum et al., 1998). Fatigue was also associated with physical and mental distress, which could lower QOL.

The strengths of this study included the multi-site design, relatively large sample size and use of standardized instruments. However, several methodological limitations should be acknowledged. First, casual relationships between fatigue and other variables could not be established due to cross-sectional design. Second, only five university nursing schools were included, and hence, our findings may not be generalizable to all nursing students in China. Third, some factors (e.g., academic pressure and social support) associated with fatigue were not assessed due to logistical reasons.

## Conclusion

Fatigue was common among nursing students in post-COVID-19 era. Considering the negative impact of fatigue on QOL and daily functioning, routine physical and mental health screening should be conducted for nursing students. Effective stress-reduction strategies should be executed to assist nursing students to combat fatigue and restore optimal health.

## Supplemental Information

10.7717/peerj.11154/supp-1 Supplemental Information 1 Raw data.

10.7717/peerj.11154/supp-2 Supplemental Information 2 Questionnaire.

## Additional Information and Declarations

### Data Availability

The following information was supplied regarding data availability: Raw data and the questionnaire are available in the Supplemental Files.
