# Supplementary material for: The prevalence of fatigue among Chinese nursing students in post-COVID-19 era
Source: PeerJ. 2021 Apr 13;9:e11154. doi: 10.7717/peerj.11154 (PMC8051357; doi:10.7717/peerj.11154)
Supplement: Supplemental Information 2 [file peerj-09-11154-s002.pdf]

Chinese Version

新冠疫情后医学生生活质量现状调查

亲爱的同学们：

您好。为了减轻新冠疫情对目前我国护理学教育的负面作用，我们需要了解本次疫情对本科学生生活质量潜在影响。这是一项北京大学、吉林大学、武汉大学、兰州大学和首都医科大学联合进行的在线调查。填写本问卷大约需要 15 分钟。这是一项匿名调查，请您按照实际情况如实填写。我们对收集的数据资料保密，只作学术研究用。

一、基本资料

1. 您所在的学校： [单选题] *

○北京大学

○吉林大学

○武汉大学

○兰州大学

○首都医科大学

2. 您的年龄：______ 周岁 [填空题] *

3. 您的性别： [单选题] *

○女

○男

4. 您是否为独生子女： [单选题] *

○否

○是

5. 您居住在： [单选题] *

○农村

○城市

6. 您的年级： [单选题] *

○一年级

○二年级

○三年级

○四年级

7.你是否参加了疫情相关志愿服务： [单选题] *

○未参加

○参加

8.您是否体验到了的疫情相关的负面经历（疫情期间经历医疗暴力、性侵犯、压力应激、 社会歧视与偏见）： [单选题] *

○否

○是

9.您是否在疫情期间频繁使用社交媒体(QQ、微信、微博、抖音等)？： [单选题] *

○否

○是

10. 本次疫情，您家庭的经济损失大吗？ [单选题] *

○无，或很小

○有损失，但不算大

○非常大;

11. 您家庭目前的经济状况： [单选题] *

○不好

○一般

○很好

12. 您目前的健康状况： [单选题] *

○不好

○一般

○很好

二、PHQ-2

请根据您最近一周的情况进行回答，如果一周内曾有波动，请以目前情况为准。

1. 做事时提不起劲或没有兴趣： [单选题] *

○完全不会

○好几天

○一半以上的天数

○几乎每天

2. 感到心情低落、沮丧或绝望： [单选题] *

○完全不会

○好几天

○一半以上的天数

○几乎每天

三、GAD-7

请根据您最近一周的情况进行回答，如果一周内曾有波动，请以目前情况为准。

1. 感觉紧张，焦虑或急切 ： [单选题] *

○完全不会

○好几天

○一半以上的天数

○几乎每天

2. 不能够停止或控制担忧： [单选题] *

○完全不会

○好几天

○一半以上的天数

○几乎每天

3. 对各种各样的事情担忧过多： [单选题] *

○完全不会

○好几天

○一半以上的天数

○几乎每天

4. 很难放松下来： [单选题] *

○完全不会

○好几天

○一半以上的天数

○几乎每天

5. 由于不安而无法静坐： [单选题] *

○完全不会

○好几天

○一半以上的天数

○几乎每天

6. 变得容易烦恼或急躁： [单选题] *

○完全不会

○好几天

○一半以上的天数

○几乎每天

7. 感到似乎将有可怕的事情发生而害怕： [单选题] *

○完全不会

○好几天

○一半以上的天数

○几乎每天

四、生活质量

1、您怎样评估您的生活质量？ [单选题] *

○极不满意

○不满意

○没有满意或不满意

○很满意

○极满意

2.您满意自己目前的健康状况吗？ [单选题] *

○极不满意

○不满意

○没有满意或不满意

○很满意;

○极满意

五、疲惫:

最能符合目前您实际疲惫程度的数字是：(   )

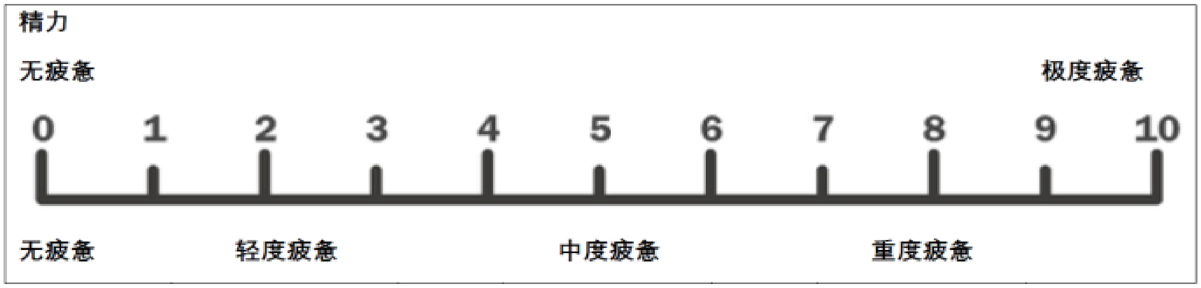

请点击下面合适的数字来完成此项的测评： [单选题] *

无疲惫 ○0 ○1 ○2 ○3 ○4 ○5 ○6 ○7 ○8 ○9 ○10 极度疲惫

六、疼痛:

最能符合目前您实际身体疼痛程度的数字是：(   )

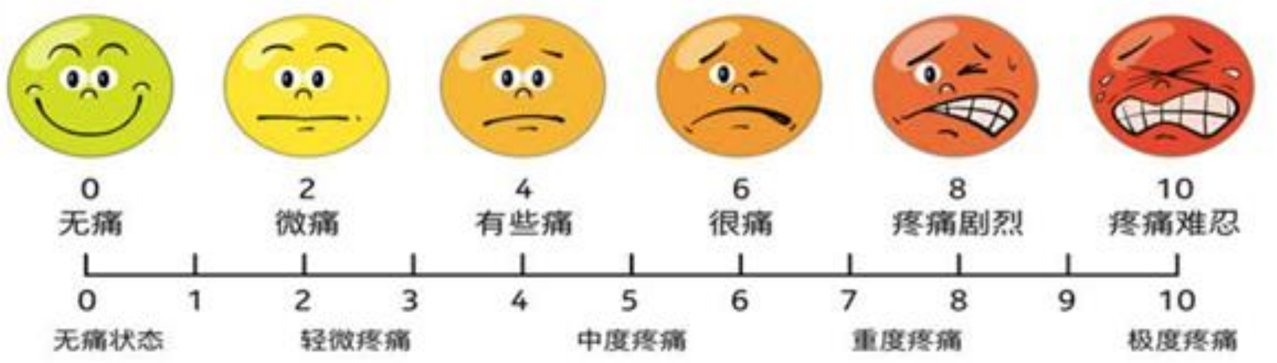

请点击下面合适的数字来完成此项的测评： [单选题] *

无痛 ○0 ○1 ○2 ○3 ○4 ○5 ○6 ○7 ○8 ○9 ○10 疼痛难忍

English Version

Study on the quality of life of medical students after COVID-19 era

Dear students,

Hello, this survey was jointly initiated by the Mental Health Committee of Chinese nursing association, and the Chinese nursing association otolaryngology branch and the University of Macau. It aims to understand the mental psychology of nursing students after the novel coronavirus epidemic. The health status provides a basis for carrying out relevant health education, services and nursing training in the future. Please fill in the questionnaires truthfully according to your situation. The questionnaires are submitted anonymously and cost nearly fifteen minutes. We will strictly protect your information and will not leak your personal information. Thank you very much for your support and participation!

Thank you!

一、Basic Information

1.Your university name is:(   )

1=Peking University   2=Jilin University   3= Wuhan University
4=Lanzhou University   5=Capital Medical University

2.Age(years)：________

3.Gender:(   )

0=Female   1=Male

4. Whether are you the only child at home? (   )

0=No   1=Yes

5. Where do you living? (   )

0=Rural   2=Downtown

6. What grade are you now? (   )

0=First year   1= Second year   2=Third year   3=Fourth year

7.Whether did you being volunteer during COVID-19 pandemic? (   )

0=No   1=Yes

8.Whether did you have negative experiences during COVID-19 pandemic (medical violence, sexual assault, stress, and stigma during the epidemic)? (   )

0=No   1=Yes

9.Whether did you frequently use of social media during COVID-19 pandemic(QQ, WeChat, Weibo, Twitter, Douyin)？ (   )

0=No   1=Yes

10.Whether did your family have a of economic loss in this epidemic? (   )

0=None, or very small   1=Yes, a little   2=Yes, very large

11. What is your family economic statue? (   )

0=No good   1=General   2=Very good

12.What is your current health statue? (   )

0=No good   1=General   2=Very good

二、PHQ-2

Over the last 1 weeks, how often have you been bothered by the following problems?

1.Little interest or pleasure in doing things (   )

0=Not at all   1=Several days   2=More than half the days   3=Nearly every day

2. Feeling down, depressed, or hopeless (   )

0=Not at all   1=Several days   2=More than half the days   3=Nearly every day

三、GAD-7

Over the last 1 weeks, how often have you been bothered by the following problems?

1. Feeling nervous, anxious, or on edge(   )

   0=Not at all   1=Several days   2=More than half the days   3=Nearly every day

2. Not being able to stop or control worrying(   )

   0=Not at all   1=Several days   2=More than half the days   3=Nearly every day

3. Worrying too much about different things(   )

   0=Not at all   1=Several days   2=More than half the days   3=Nearly every day

4. Trouble relaxing(   )

   0=Not at all   1=Several days   2=More than half the days   3=Nearly every day

5. Being so restless that it’s hard to sit still(   )

   0=Not at all   1=Several days   2=More than half the days   3=Nearly every day

6. Becoming easily annoyed or irritable(   )

   0=Not at all   1=Several days   2=More than half the days   3=Nearly every day

7. Feeling afraid as if something awful might happen(   )

   0=Not at all   1=Several days   2=More than half the days   3=Nearly every day

四、Quality of life

1. How would you rate your quality of life?(   )

   0=Very poor   1= Poor   2= Neither poor nor good   3= Good   4= Very good

2.How satisfied are you with your health? (   )

   0=Very dissatisfied   1=Dissatisfied   2=Neither satisfied nor dissatisfied

   3= Satisfied   4=Very satisfied

五、Fatigue:

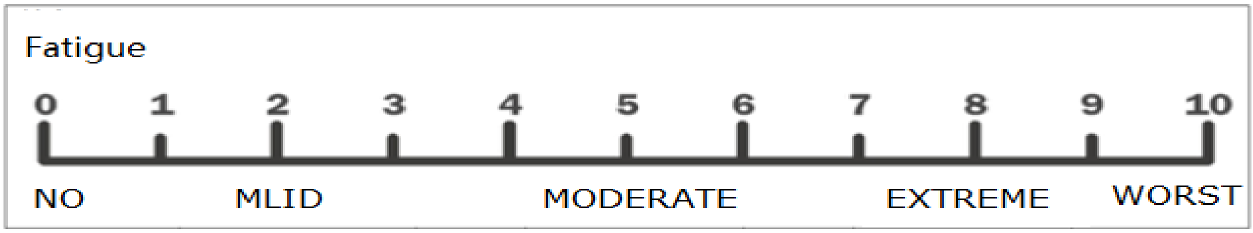

Please click on the appropriate number to complete the assessment for this item: (   )

☐0 ☐1 ☐2 ☐3 ☐4 ☐5 ☐6 ☐7 ☐8 ☐9 ☐10

六、Pain:

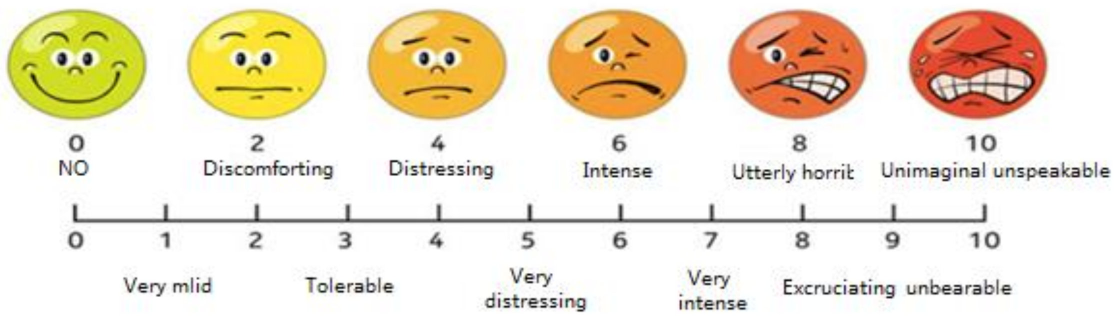

Please click on the appropriate number to complete the assessment for this item: (   )

☐0 ☐1 ☐2 ☐3 ☐4 ☐5 ☐6 ☐7 ☐8 ☐9 ☐10
